# TSPO expression in brain tumours: is TSPO a target for brain tumour imaging?

**DOI:** 10.1007/s40336-016-0168-9

**Published:** 2016-03-22

**Authors:** Federico Roncaroli, Zhangjie Su, Karl Herholz, Alexander Gerhard, Federico E. Turkheimer

**Affiliations:** Wolfson Molecular Imaging Centre, The University of Manchester, 7 Palatine Road, Withington, Manchester, M20 3LJ UK; Department of Neuroimaging, IoPPN, King’s College London, London, UK

**Keywords:** Translocator protein, Brain tumours, PET imaging

## Abstract

Positron emission tomography (PET) alone or in combination with MRI is increasingly assuming a central role in the development of diagnostic and therapeutic strategies for brain tumours with the aim of addressing tumour heterogeneity, assisting in patient stratification, and contributing to predicting treatment response. The 18 kDa translocator protein (TSPO) is expressed in high-grade gliomas, while its expression is comparatively low in normal brain. In addition, the evidence of elevated TSPO in neoplastic cells has led to studies investigating TSPO as a transporter of anticancer drugs for brain delivery and a selective target for tumour tissue. The TSPO therefore represents an ideal candidate for molecular imaging studies. Knowledge of the biology of TSPO in normal brain cells, in-depth understanding of TSPO functions and biodistribution in neoplastic cells, accurate methods for quantification of uptake of TSPO tracers and pharmacokinetic data regarding TSPO-targeted drugs are required before introducing TSPO PET and TSPO-targeted treatment in clinical practice. In this review, we will discuss the impact of preclinical PET studies and the application of TSPO imaging in human brain tumours, the advantages and disadvantages of TSPO imaging compared to other imaging modalities and other PET tracers, and pathology studies on the extent and distribution of TSPO in gliomas. The suitability of TSPO as molecular target for treatment of brain tumours will also be the appraised.

## Introduction

PET imaging has increasingly been explored in the field of neuro-oncology for pre-operative prediction of tumour grade, monitoring of disease progression and response to treatment, and to acquire information about the expression and distribution of molecules that can be targeted for treatment [1].

The mitochondrial translocator protein (TSPO) is one of the PET imaging target molecules that has been investigated in the last two decades [2, 3]. The TSPO offers several advantages in the study of brain tumours, because its expression is increased in tumour cells but low in the normal brain and the expression can be visualized and quantified with PET imaging [4]. The first studies looking at the density and distribution of TSPO with autoradiography in mouse and postmortem human brain sections date over two decades [5–8]. The first preclinical and clinical PET imaging studies suggested an application of TSPO imaging in clinical practice [9, 10], but during the 20 years that followed, research almost exclusively focused on gliomas despite the great variety, heterogeneity and complexity of tumours of the central nervous system (CNS).

This review aims to appraise the literature and discuss the advantages and challenges of investigating the TSPO as a target of molecular imaging for CNS tumours.

### The complexity and heterogeneity of CNS tumours

An accurate classification of CNS tumours is essential for the planning, development and interpretation of molecular imaging studies, validation of tracers, correlations with outcome, response to treatment and translation of preclinical studies into clinical application.

Tumours of the CNS comprise more than 120 types. They are classified and graded according to the World Health Organization (WHO) (WHO Blue Book) [11]. The WHO blue book represents a set of consensus guidelines aiming at establishing criteria for clinical practice. The Blue Book distinguishes clinico-pathological entities, variants and histological patterns. An entity has distinctive morphology, location, age distribution and biologic behaviour. Variants can be identified histologically and have some relevance for clinical outcome, but are mostly parts of a defined entity. Patterns of differentiation are visually identifiable by nature without distinct clinical or pathological features [11].

The WHO Blue Book also recommends a histological grade. Grading is a ‘malignancy scale’ intended to predict the natural biological behaviour of a neoplasm. In general, grade I applies to lesions with low proliferative potential and often cured with surgery. Grade II defines tumours that are usually infiltrative and often recur despite their low growth potential. Some grade II tumours progress to higher grade. Grade III defines lesions with histological evidence of malignancy and WHO grade IV is assigned to tumour associated with rapid progression and a fatal outcome. The WHO grade is not an absolute predictor of outcome. Other factors such as age, performance status, location, extent of surgical resection and genetic alterations are relevant to an overall estimate of prognosis [11].

An in-depth understanding of the molecular and genetic basis of CNS tumours has led to a significant improvement of diagnostic accuracy and ability to estimate the outcome and to guide the treatment of several tumour entities [12]. In addition to the histotype and grade, molecular and genetic features should be considered in molecular imaging studies that are designed to stratify patients or identify measures of outcome.

### Preclinical models: in vivo imaging models and in vitro studies

PET imaging in small animals became available at the end of the 1990s [13, 14], about 10 years after the initial studies on TSPO. The recent introduction of small animal devices with high resolution and the possibility of co-registering PET and MRI have boosted preclinical research on CNS tumours [14]. So far, preclinical imaging studies on TSPO in CNS tumours have focused mostly on assessing the suitability of second-generation TSPO ligands in glioma models.

Buck et al. first tested [^18^F] PBR06 in rat implanted with the C6 glioma cell line [15]. The signal was fourfold higher than in the normal brain and its accumulation in neoplastic tissue closely reflected TSPO protein levels measured in the tissue. The authors attempted to fit a compartmental model (the classic two-tissue compartment model with four rate constants) to the dynamic PET data, but did not obtain meaningful estimates of the volume of distribution (Vt) and had to adopt simplified graphical approaches to obtain stable Vt estimates. This aspect of their work is relevant, because it highlights the complexity of the quantification for TSPO tracers. The heterogeneity of cellular distribution of TSPO that is already present in the normal brain (vascular, parenchyma) is compounded in and around tumours by the variability of vascularization, perfusion and cellularity, typical of high-grade gliomas. Interestingly, Buck and colleagues reported slightly higher uptake in the peripheral regions of the tumour and suggested that PET imaging can identify the proliferative and infiltrative components of gliomas. This result contrasts with studies based on serial stereotactic biopsies that demonstrated decreased proliferation in the infiltrative edge of gliomas compared to the tumour centre, while adhesion molecules were more abundant at the periphery to confer a migratory, infiltrative phenotype [16]. The same group at the Vanderbilt University subsequently evaluated [^18^F]DPA-714 in a similar rat model [17]. Like [^18^F]PBR06, [^18^F]DPA-714 uptake correlated with TSPO protein levels in the tissue, but a head-to-head comparison of the two tracers showed better retention of [^18^F]DPA-714. More recently, they generated the novel ligand [^18^F]VUIIS1008 showing 36-fold higher affinity than its parent compound [^18^F]DPA-714 [18]. [^18^F]VUIIS1008 had greater tumour avidity, lower uptake in the normal brain, better tumour-to-background ratio and higher binding potential than [^18^F]DPA-714. However, the perfusion rate of [^18^F]DPA-714 (K1) was much higher than [^18^F]VUIIS1008, suggesting that more tracer crossed the blood brain barrier (BBB) while the affinity for TSPO was higher for [^18^F]VUIIS1008. This evidence indicates that [^18^F]DPA-714 performed slightly better if the simple SUVs (integrated radioactivity) was used as a measure of uptake. A kinetic modelling should have been applied to see an improved ratio for [^18^F]VUIIS1008. [^18^F]VUIIS1008 and its parent compounds have limitations such as the still too high accumulation in the normal brain that could obscure gliomas with low TSPO expression. In addition, in vivo metabolism requires a corrected plasma input function for modelling, long dynamic PET acquisitions and compartmental analysis that are not always possible in a routine clinical practice.

[^18^F]DPA-714 was also evaluated by Winkeler and colleagues in an intracranial rat glioma model where the 9L glioma cell line was implanted into three different rat strains, including Fischer, Wistar and Sprague–Dawley [19]. In keeping with the previous study, the results showed satisfactory uptake at the site of glioma as compared to the contralateral brain hemisphere and cerebellum. Immunostains on tissue demonstrated that TSPO was mostly restricted to tumour cells with a contribution of glioma-associated microglial/macrophages (GAMs), but not reactive astrocytes around the lesion. ^18^F-DPA-714 was also tested in the same model to monitor the efficacy of erufosine on gliomas [20].

Finally, the recent preclinical study by Buck and colleagues is of potential relevance to the progress of molecular imaging in human gliomas [21]. They used human low- and high-grade glioma samples established orthotopically in NOD/SCID mice and then passaged into athymic rats. Unlike conventional models, this xenotransplants allowed evaluation of invasion along the white matter tracts into the hemisphere contralateral to the tumour site. [^18^F]PBR06 showed uptake beyond the region identified with MRI T2-weighted images and highlighted the infiltrative growth in the contralateral hemisphere. This result suggests that TSPO PET imaging could potentially serve as a non-invasive tool to evaluate the disease extent. Similar to our previous study [22], [^18^F]PBR06 imaging reflected TSPO expression in neoplastic cells rather than GAMs.

### In vitro models

The TSPO has been widely studied in glioma cell lines, but it is still unclear whether this molecule has causative or contributory role in tumorigenesis or whether up-regulation represents an epiphenomenon accompanying cancer [23]. Providing a detailed summary of preclinical research is beyond the scope of this review; we will therefore discuss studies that may contribute to a more accurate interpretation of molecular imaging results.

The work by Bode and colleagues [23] can be relevant to PET imaging, as it suggests that the down-regulation of TSPO correlates with an aggressive phenotype. The authors exploited the human glioma cell line U118MG to look at the effects of TSPO knockdown and exposure to increasing doses of PK11195 on tumourigenesis. *Tspo* gene silencing resulted in a reduction in adhesion molecules, increased migration in the Boyden chamber, bigger lesions with ragged margin when implanted in chorioallantoid membrane, reduced cell death, increased proliferation, decreased contact inhibition and increased angiogenesis. Implants of TSPO-negative U118MG cells in xenografts were much larger than controls. Bode and colleagues’ data contrasts with the work discussed above [21] and our study [22] that observed high TSPO level in the infiltrative front of gliomas. Such discrepancies reiterate the fact that different preclinical models and studies based on clinical samples can produce inconsistent results.

The residence time, which is defined as the ratio between injected activity and accumulated ligand in a target organ, is an important parameter to characterize radiolabeled ligands and even more so when imaging ligands that need to be exploited as anti-cancer drugs. PET imaging studies have examined the residence time of TSPO tracers in the brain of healthy adults and children [24] and subjects with neuroinflammation [25], but little is known of the effect of long-term stable occupancy. To date, only one preclinical study has addressed this question [26]. Costa and colleagues used the synthetic compound irDE-MPIGA that is designed to bind TSPO covalently and permanently at nanomolar doses, alongside the reversible TSPO ligand PIGA to investigate the effects of stable and transient residence time in the human glioma cell line U87MG cells. A nanomolar dose of irDE-MPIGA induced the early phases of apoptosis, but 24 h treatment caused only a 40 % abolition of the [^3^H]PK11195 binding and RT-PCR analysis showed that TSPO mRNA levels increased indicating restoration of TSPO synthesis. These results suggest that direct pharmacological targeting of TSPO may not be effective in gliomas.

Although unrelated to CNS tumours, two studies have provided valuable data to TSPO imaging in neuro-oncology. The experiments by Klubo-Gwiezdzinska and colleagues [27] in thyroid carcinoma cell lines knocked down for TSPO showed sixfold increase of TSPO transcript following exposure to valproic acid. Induction of TSPO by valproic acid was also associated with increased sensitivity to oxidative stress after challenge with H_2_O_2_. In this model, proliferation, cell viability and migration were not different between TSPO-rich and TSPO-deficient cells and were unaffected by silencing of the *TSPO* gene. By exploiting the dataset available in Geo Profiles (http://www.ncbi.nlm.nih.gov/geoprofiles), the authors observed a reduction in TSPO expression in aggressive and recurrent tumours. The data presented in this study can be relevant to patients with CNS tumours. Evidence-based guidelines suggest that levetiracetam is the best first-line agent for treatment of seizures in patients with low-grade glioma, but approximately one-third of such patients have refractory epilepsy requiring other agents including valproic acid [28]. Similar to Bode et al. [23], the evidence of a positive correlation between low TSPO and aggressive behaviour suggests that increased binding of TSPO ligands to tumour tissue is not always a prognostic indicator of poor outcome as previously widely believed.

Little is known about the regulation of TSPO synthesis. Gene amplification does not appear to be sufficient to explain the increased TSPO in cancer cells. Batarseh and colleagues [29] published the first in-depth description of the human *TSPO* gene promoter and its transcriptional regulation. With the breast cancer cell lines MCF-7 and MDA-MB-231 performing as models of high and low *TSPO* gene promoter methylation, they demonstrated that promoter silencing was not the only epigenetic mechanism regulating the expression and that the transcription factors Sp1, Sp3 and Sp4 also contribute to the overall levels of expression. The authors also proposed that multiple potential transcription factor binding sites are represented in the *TSPO* gene promoter, leading to the suggestion of a different regulation of this gene in different cell types and tissues. The histone deacetylase inhibitor TSA induced *TSPO* promoter activity and reduced the methylation signature, suggesting that epigenetic modification of chromatin and DNA can contribute to the regulation of promoter activity. An effort should be made to clarify epigenetic regulation of *TSPO* gene in CNS tumour entities before embarking on TSPO imaging studies.

### TSPO and brain metastases

Brain metastases are the most common intracranial neoplasms. Their prevalence outnumbers primary CNS tumours by around ten times and represents a leading cause of death in cancer patients [30, 31]. The incidence of brain metastases has been increasing steadily over the last decade as a result of more effective treatments for primary cancer, thereby increasing the duration of patient survival. All types of cancer can potentially metastasize to the CNS, with lung, breast and renal carcinomas and melanomas representing more than 90 % of deposits [30]. Despite the extensive screening of TSPO in cancer types outside the CNS [32] and the association of high TSPO with disease progression and diminished survival [33], TSPO in brain metastases from solid cancer is still unexplored territory.

The only brain metastasis model has recently been published by O’Brien and colleagues [34]. The mouse mammary carcinoma 4T1 known to express negligible TSPO was injected into the brain or heart of the animals to explore the early changes occurring in astrocytes and microglia. To do so, they used ^123^I-DPA713 SPECT imaging, immunohistochemistry and ex vivo autoradiography. Following both intracerebral and intracardiac injection, the authors observed an association between metastases and reactive astrocytosis in the surrounding tissue that persisted across the 28-day time course. Astrocytosis positively correlated with the volume of the deposit in intracerebral implants with a constant ratio between tumour cells and reactive astrocytes described. In contrast, the ratio of reactive astrocytosis and tumour area increased significantly following intracardiac injection, which results in haematogenous spread. Both models showed microglial activation at the sites of the deposit. Astrocytes and microglia expressed TSPO, but the degree of expression varied between the different types of implants. The conclusion that microglial and astrocytic TSPO represent a surrogate marker of metastatic deposit when neoplastic cells are negative or express low TSPO level is relevant to imaging studies. Interestingly, ex vivo autoradiography ^123^I-DPA713 highlighted glial activation in the intracardiac model, but in vivo SPECT failed to demonstrate individual metastases. The reason for such a discrepancy is unclear.

The heterogeneity of cell types expressing TSPO in metastatic deposits was also proved by Zheng and colleagues [35] after subcutaneous inoculation of the high TSPO MDA-MB-231 breast cancer line and low TSPO MCF-7. The tissue was examined autoradiographically with the TSPO ligand [^18^F]DPA-714 and by immunohistochemistry. Both neoplastic cells and macrophages contributed to the overall TSPO load. TSPO did not directly correlate to the tumour growth rate, metastatic potential or drug responsiveness. Unlike gliomas, the majority of macrophages associated with metastases were TSPO positive, but differences existed among the cell types, with one having the lowest macrophage density and one triggering the highest macrophage response. Lymphocytes and neutrophils were also shown to contribute to TSPO signal.

### The TSPO in gliomas: clinical studies and methodological consideration

The quantification of TSPO in PET imaging studies is challenging and brain tumours are not an exception. Preclinical models have not been successfully translated into clinical studies, due to the differences between human and animal biology and the fact that animal studies are performed in controlled conditions. An overview of methods for quantification of binding potentials has recently been provided by Hinz [36].

Quantification of binding potentials generally requires an “input function”, providing an estimate of kinetic tracer delivery to the tissue. It can be achieved by multiple arterial blood samples with correction of tracer activity due to metabolites, but this is not always practically possible. Alternatively, the time–activity curve (TAC) of an unaffected region or tissue can be used as a reference under the general assumption that the tissue selected as reference has very low TSPO expression, while perfusion is similar to tumour tissue. Another option is the use of pseudo-reference regions derived from kinetic tracer analysis in brain tissue, the “supervised cluster input function” [37].

Reference region approaches may have limitations in patients with CNS tumours. No data are available on the extent of microglial TSPO density in the tissue distant from the tumour epicentre. Patients with intrinsic brain tumours often suffer from epilepsy, which is known to trigger widespread microglial activation [38]. Badie and Schartner [39] documented microglial activation in the tissue distant from the tumour in their glioma mouse model, indicating widespread inflammation. They observed that macrophages were concentrated within the glioma tissue and its periphery, while microglia were distributed throughout the CNS including the hemisphere opposite to that of the implanted tumours. The authors suggested that such a distinctive distribution could be explained by the differences in the migratory capacity and possibly the CNS immune surveillance function of microglia, as compared to macrophages.

The TSPO is ubiquitously expressed in endothelial and smooth muscle cells of vessels and such expression might potentially obscure the signal from neoplastic cells. Binding to vascular TSPO appears to be particularly relevant for tracers with very high affinity, such as 11C-PBR28 and other “second-generation” tracers. It may require the introduction of a specific irreversible vascular binding compartment into the kinetic model [40], a problem which has not been encountered in preclinical studies. Also, there is no precise information on TSPO in newly formed vessels in high-grade astrocytomas and oligodendrogliomas and on whether the altered conformation of normal vessels that are distorted by tumour growth might affect the modelling. The polymorphism rs6971 leading to the amino acid substitution A147T represents an additional problem, as it causes a variation in the binding affinity of new compounds [41]. Genotyping for this polymorphism in patients with CNS tumours would therefore be required before enrolment in TSPO imaging studies. Finally, methodological approaches and interpretation of results are hampered by the lack of understanding of TSPO functions and the inconsistency of results obtained in in vitro and in vivo experiments. To stress such uncertainty, Selvaraj and Stocco [42] even questioned the word ‘ligand’ for TSPO studies. They remind the readers that ‘ligand’ refers to a small molecule that binds to a target and elicits or modulates a specific agonist or antagonist response directly associated with the function of the target molecule. The evidence that PK11195 can bind the F0F1 ATP synthase [43] within the hydrophobic chain region of lipid membranes [44] adds further complexity.

**Fig. 1:**
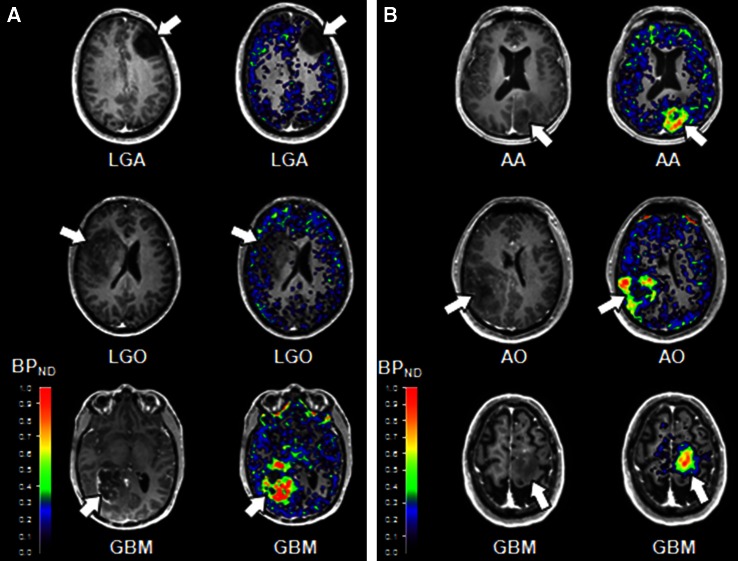
**a** Co-registered and fused post-contrast T1-weighted MRI (*greyscale*) and parametric BP_ND_ images (spectrum colour scale) of representative cases of LGA, LGO and HGG; note that BP_ND_ is low in LGA, whilst high BP_ND_ foci are found in LGO and high BP_ND_ areas in GBM. **b** Co-registered post-contrast T1-weighted MRI and parametric BP_ND_ images in HGGs showing little or no contrast enhancement, and high [^11^C]-(*R*)-PK11195 binding within the tumours. The colour bars denote BP_ND_ values; the *white arrows* indicate tumour location. *LGA* low-grade astrocytoma, *LGO* low-grade oligodendroglioma, *AA* anaplastic astrocytoma, *AO* anaplastic oligodendroglioma, *GBM* glioblastoma, *HGG* high-grade glioma

**Fig. 2:**
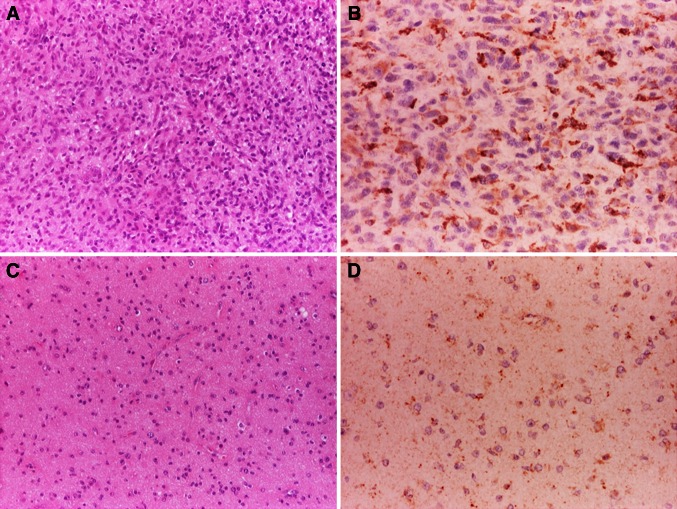
Analysis of the tissue from the surgery of patients who had [^11^C]-(*R*)-PK11195 demonstrates high TSPO expression in high-grade astrocytomas; the images document a case of anaplastic astrocytoma, WHO grade III (**a** haematoxylin-eosin; ×10) and the extent of TSPO expression in neoplastic cells (**b** TSPO immunoperoxidase; ×20); TSPO expression is much lower in low-grade astrocytoma (WHO grade II) (**c** haematoxylin–eosin; ×10), but retained in the infiltrative component (**d** TSPO immunoperoxidase; ×20)

**Fig. 3:**
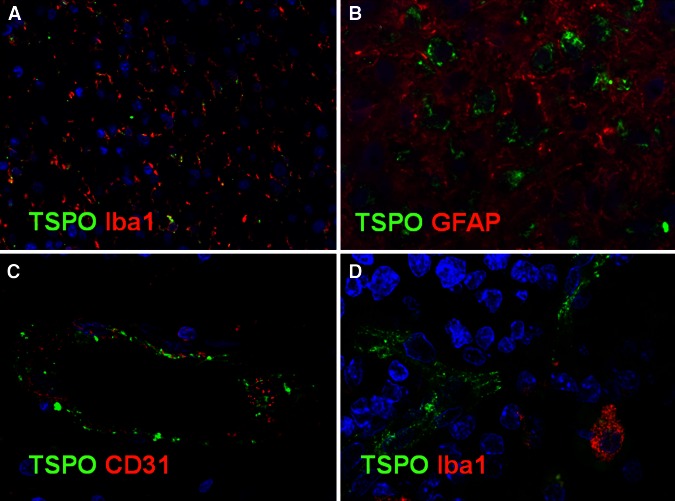
The double immunofluorescence staining for TSPO and the microglial/macrophage marker Iba1 showed that only some microglial cells were TSPO positive (**a** anaplastic astrocytoma; TSPO* green Alexa Fluor 546* and Iba1* red Alexa Fluor* 488; ×10); there was no colocalization of TSPO* green Alexa Fluor* 546 and GFAP-positive reactive astrocytes (**b** anaplastic astrocytoma; TSPO* green Alexa Fluor* 546 and Iba1* red Alexa Fluor* 488; ×20); endothelial cells express TSPO in a leptomeningeal artery (**c** low-grade astrocytoma; TSPO* green Alexa Fluor* 546 and CD31* red Alexa Fluor* 488; ×20) and in a capillary (**d**, low-grade oligodendroglioma; TSPO * green Alexa Fluor* 546 and Iba1* red Alexa Fluor* 488; ×40)

Despite the limitations discussed above, our work [22, 44] proved the suitability of [^11^C]-(R)-PK11195 in molecular imaging of low- and high-grade gliomas, and its potential to identify anaplastic transformation (Fig. 1). Binding was modelled using the cerebellum as the reference, providing more robust results in this patient group than the supervised cluster input function. Using this approach, negative binding potentials were recorded in most WHO grade II gliomas, indicating that TSPO binding in the cerebellum was higher than in those tumours. We observed only limited impact of the blood–brain barrier disruption on the profiles of tracer kinetics and our results indicated that TSPO PET could be more sensitive than structural imaging in the early detection of anaplastic transformation. By quantifying the intensity and surface of TSPO-positive cytoplasm in the tissue removed at surgery from the same patients who underwent ^11^C-(R)-PK11195 PET imaging, we could correlate tissue expression with imaging data and determine the threshold below which ^11^C-(R)-PK11195 uptake does not appear to be detectable at imaging (Fig. 2a, b). Notably, cells in the infiltrative component were as positive as cells in the tumour centre (Fig. 2c, d). We also proved that TSPO is predominantly expressed in neoplastic cells, with GAMs only partially contributing to PET signal and, similar to Winkeler et al. [19], we found no expression in reactive astrocytes (Fig. 3a, b). Oligodendrogliomas demonstrated higher ^11^C-(R)PK11195 binding than expected from tissue analysis. Their vascular density is evident in rCBV maps and the tortuosity of vessels [45] may account for such discrepancy (Fig. 3c, d). In addition, the higher microglial TSPO and perhaps a varying affinity across oligodendrogliomas may contribute to the distinct kinetic.

Three other PK11195 PET studies have been conducted in a small number of glioma patients. Juncket al. [8] performed the first [^11^C]-PK11195 PET study in human astrocytomas. They showed increased uptake in the late images in eight out of ten patients and suggested that such accumulation of [^11^C]-PK11195 represented saturable high-affinity binding to TSPO. However, they did not investigate the cellular sources of the binding, nor did they observe any correlations between tumour grade and tumour radioactivity or in the ratio of tumour over normal grey or white matter. The low specific radioactivity of [^11^C]-PK11195 in some cases and the use of racemic tracer might have led to underestimation of the binding. Subsequently, Pappata et al. [46] demonstrated in a patient with GBM that [^11^C]-PK11195 binding was twofold higher in the tumour than in the normal grey matter and that 30 % of binding in the tumour could be displaced by a large excess of unlabelled ligands. Their finding confirmed that [^11^C]-PK11195 retention in the tumour was in part due to specific binding. Again, the cellular source of the binding was not mentioned. Later on, in a PET investigation of two patients with anaplastic astrocytoma, Takaya et al. [47] pointed out that [^11^C]-*R*-PK11195 binding was markedly lower in the tumours than in the contralateral grey matter. Tissue examination revealed that GAMs failed to express TSPO, although both tumours contained a substantial number of microglia.

### TSPO and SPECT

The availability of radiolabelled TSPO ligands for SPECT could potentially allow the clinical application on a larger scale because of its relatively low cost [48]. Ro5-4864 was radiolabelled with iodine-125 to yield a SPECT tracer [49]. Subsequently, a ^123^I-labelled analogue of PK11195 displayed selective binding to the TSPO in human GBM cells [50]. A [^123^I]-iodo-PK11195 is currently used in human SPECT as it has sufficient specific activity for quantitative studies [51]. Feng et al. [52] scanned four patients with GBM and six patients with stroke, aiming to streamline the best ^123^I-CLINDE quantification approaches for clinical studies. They reported a 1.8- to 3.4-fold higher distribution volume of the tracer in the GBMs compared with that in the cerebellum, and no significant changes in ^123^I-CLINDE binding parameters in areas of gadolinium leak. They concluded that ^123^I-CLINDE binding was not importantly affected by BBB disruption, and ^123^I-CLINDE SPECT could be used for quantitative assessment of TSPO expression in vivo.

The SPECT study by Jensen and colleagues [53] is of interest. They addressed the clinical application of TSPO imaging in GBM patients by comparing the second-generation tracer ^123^I-CLINDE with ^18^F-FET PET and contrast-enhanced structural MRI. The authors studied three patients with advanced GBM previously genotyped for the rs6971 polymorphism. None of the subjects received anti-angiogenesis drugs for 6 weeks before the scans or had any radio-chemotherapy between scans. They observed limited volumes of interest (VOI) overlap, indicating that imaging of ^18^F-FET and TSPO binding reflects different aspects of the tumour. In addition, ^18^F-FET uptake was shown to be not entirely specific to glioma cells, although it should not bind to GAMs. In this respect, the authors suggested that the TSPO-negative/^18^F-FET–positive areas represent reactive astrocytosis. ^18^F-FET VOIs gave a better overlap with contrast-enhanced VOIs than ^123^ICLINDE VOIs and the signal of ^123^I-CLINDE was not increased in the areas of contrast enhancement where the BBB was disrupted. Finally, the VOIs of increased ^123^I-CLINDE at baseline appeared to be a good predictor of tumour progression, suggesting a potential for TSPO imaging to provide clinically relevant information regarding areas of proliferation.

### What have we learnt from pathology?

The extent and distribution of TSPO in the tissue of human gliomas has been evaluated in a few studies.

It is now 20 years since Miettinen et al. [54] first documented an association between TSPO level and WHO grade in a series of 86 surgically removed adult supratentorial astrocytomas. Tumours were classified and graded according to the 1993 edition of the WHO Blue Book; on WHO grade II and III may not therefore be directly comparable with more recent studies. The authors showed that transcript and protein are undetectable in the normal brain and found negligible expression in pilocytic astrocytomas. With the caveat of classification, grade II and III tumours showed moderate expression without significant difference between the two grades and with some cases being negative. Expression in GBM was the highest and uniform in all but 5 of the 37 cases examined. Similar to our recent study, Miettinen et al. proved that TSPO increased irrespective of cell density and found that increased protein levels depended on increase in gene expression. Vlodawsky et al. [55] found similar levels and distribution of expression in their series of 130 astrocytomas classified according to the 1999 edition of the WHO classification. There was no detectable expression in the normal control brains or in the normal brain tissue that was present in surgical samples. Pilocytic astrocytomas were virtually negative. Interestingly, none of these studies mentioned endothelial TSPO or its expression in microglia and macrophages. An interesting discovery of endogenous TSPO ligand diazepam-binding inhibitor in the same cells expressing TSPO suggests an autocrine loop [54].

Both studies documented a statistically significant association between TSPO levels and cell proliferation determined with MIB-1, leading to the suggestion that TSPO activity is somehow related to the latter. However, the effect of tumour grade was not considered in the analysis. Vlodawsky and colleagues also found a linear correlation between TSPO expression and number of cells in apoptosis with an increase of both in the cells lining necrotic areas. Though interesting, this observation did not consider the presence of macrophages at the edge of the necrotic tissue; which can be TSPO positive and present to scavenge apoptotic cells [56]. In addition, the macrophages themselves can undergo apoptosis as shown in atherosclerotic plaques [57].

Both studies observed a positive and significant association between TSPO expression, WHO-designated tumour grade and survival. To understand if the association between TSPO expression and patient survival simply reflected the association with WHO grade, Mittienen et al. [54] looked separately at the WHO grade II astrocytomas group and confirmed that lesions with higher TSPO had a tendency for shorter survival time. Analysis of the GBM group did not reveal any difference. Results on low grades are interesting, but based on few cases and potentially flawed by the grading of these lesions based on the 1993 criteria that did not allow reliable distinction between pilocytic and diffuse astroctyomas.

Buck et al. [21] corroborated their preclinical work with the evaluation of TSPO in cohort of low- and high-grade astrocytomas using immunohistochemistry on tissue microarrays. The extent and distribution of expression were similar to the previous studies. They found the TSPO confined to the cytoplasm with only occasional nuclear expression.

Our recent results [22] are in line with the studies discussed above. We observed cytoplasmic, granular immunolabelling for TSPO consistent with mitochondrial localization. TSPO-positive cells were evenly distributed throughout the tumour tissue as well as in its peripheral, infiltrative component as seen by Buck and colleagues in their preclinical model [21]. The intensity of TSPO immunolabelling ranged from weak and limited to a perinuclear cytoplasm in low grades to intense in the entire cytoplasm anaplastic astrocytomas and GBMs. As mentioned before, only a proportion of GAMs were positive and no expression was observed in reactive astrocytes.

### Does TSPO offer advantages over other imaging modalities?

The question on whether TSPO molecular imaging offers any advantages over structural, physiological and metabolic imaging, and over other existing molecular imaging modalities, remains open.

The results from preclinical and clinical studies seem to suggest an advantage of TSPO PET imaging over structural and physiological MRI in detecting early transformation and the infiltrative edge of gliomas. Longitudinal studies are nevertheless necessary to confirm this suggestion and investigate the potential of PET in discriminating between disease progression and radiation necrosis.

TSPO imaging may also offer advantages over [^18^F]FDG PET as image interpretation is not affected by high uptake in the normal brain. Future studies are required to compare TSPO imaging with amino acid PET. Amino acid tracers, such as [^11^C]MET, [^18^F]FET and [^18^F]-FDOPA, depend on an active transport through the endothelium which is already increased in most low-grade gliomas in the absence of BBB disruption [58]. Interestingly, FDG and amino acid tracers also demonstrate higher uptake in oligodendrogliomas than in astrocytomas of the same grade [59, 60].

### The TSPO in the treatment of brain tumours

It is our opinion that molecular imaging can play a role in developing personalised treatment strategies for patients with CNS tumours by exploiting the substantially increased levels of TSPO in high-grade gliomas compared to the normal brain. However, we agree with Selvaraj and Stocco [42] that a considerable effort is still necessary to untangle the functions of TSPO before considering the TSPO as a primary treatment target.

The use of anticancer drug conjugated to TSPO ligands was suggested for selective brain delivery, particularly in lesions where the BBB is preserved [61]. Polyethylene glycol-phosphatidyl ethanolamine micelles [62] and dendrimers loaded with chemotherapeutics and coated with TSPO ligands [63] were also proposed to enhance the delivery in tumour tissue. The supposed role of TSPO in maintaining and reestablishing cell homoeostasis following oxidative stress could also be exploited in TSPO-positive tumours to increase radio and/or chemosensitivity to compounds that are routinely used in the treatment of patients with CNS cancer. However, caution should be exercised while planning studies that target TSPO, due to its widespread expression outside the CNS and adrenals, kidney liver and thyroid, in particular.

The development of nanoparticles and vehicles that could exploit TSPO to increase the bioavailability and selective delivery of radiosensitizers into tumour tissue and enhance the effect of conventional fractionated radiation seems promising. In fact, selective target of neoplastic cells with sparing of normal tissue, recognition of intracellular targets and stability of delivered chemical components remain one of the most difficult challenges in the clinical application of radiosensitizers that have not completely been resolved [64–66].

## Conclusion

Research on TSPO in CNS tumours is still at its early phase. The results obtained in gliomas are promising and suggest that TSPO imaging can predict anaplastic transformation and progression, allow better delineation of the infiltrative component and stratification of patients in view of individualized treatment (see highlights). Research should address the understanding the functions of TSPO in human CNS tumours and the evaluation of the extent of expression in the various entities.
